# Unexpectedly Simultaneous Increase in Wavelength and Output Power of Yellow LEDs Based on Staggered Quantum Wells by TMIn Flux Modulation

**DOI:** 10.3390/nano12193378

**Published:** 2022-09-27

**Authors:** Zhenxing Lv, Xiaoyu Zhao, Yuechang Sun, Guoyi Tao, Peng Du, Shengjun Zhou

**Affiliations:** 1 Center for Photonics and Semiconductors, School of Power and Mechanical Engineering, Wuhan University, Wuhan 430072, China; 2 The Institute of Technological Sciences, Wuhan University, Wuhan 430072, China

**Keywords:** bandgap-engineered quantum well, yellow LED, unexpected optoelectronic properties, TMIn flux modulation, long-wavelength optoelectronic device

## Abstract

Pursuing efficient long-wavelength InGaN LED has been a troublesome issue to be solved, which forms interesting subjects for fundamental research, but finds also motivation in extensive applications. Here, we investigate the effect of TMIn (trimethylindium) flux variation for growing bandgap-engineered staggered quantum wells (QWs) on corresponding LED properties and demonstrate the unexpectedly simultaneous increase in light output power (LOP) and emission wavelength. At 20 mA, LEDs based on staggered QWs grown under low flux show an increase of 28% in LOP and longer wavelength compared to that under high flux. The experimental results reveal that TMIn flux affects crystalline quality and indium composition of epilayers. Under high TMIn flux, high in-plane strain exists between adjacent layers, accompanied by the composition pulling effect, which reduces indium incorporation for the following staggered QW growth and hinders realization of yellow light emission. According to simulation results, low-flux-grown staggered QWs contribute to increased carrier wavefunction overlap as well as enhanced electric field. Notably, the former enables high LOP, while the latter results in emissions towards long wavelength, promising to solve an ever-present concern that LED performance deteriorates with increasing emission wavelength. Therefore, this work shows great significance in thoroughly understanding growth conditions for bandgap-engineered staggered QW structures, which offers a facile solution to achieve efficient long-wavelength optoelectronics devices.

## 1. Introduction

III-nitride light-emitting diodes (LEDs) have been considered as promising candidates for next-generation solid-state lighting and full-color displays due to their distinct advantages of high brightness, long lifetime and low energy consumption [1,2,3,4,5]. Profiting from the wide and tunable bandgap, III-nitride alloys could be adjusted to emit a spectrum spanning from deep ultraviolet to infrared [6,7]. InGaN-based LEDs generally grown along the [1] polar direction suffer from the quantum-confined Stark effect (QCSE) because of the existing intrinsic electric field across quantum wells (QWs) [8]. This separates the electron-hole wavefunction towards the opposite direction, thereby reducing internal quantum efficiency. Such negative influence related to the QCSE becomes more pronounced for longer-wavelength devices that require higher indium composition in QWs, which is known as “green-yellow gap” phenomenon [9]. Compared to state-of-the-art blue LEDs, which exhibit high external quantum efficiency up to 84.3% [10], long-wavelength LED performance remains unsatisfactory. With the increasing emission wavelength, the efficiency of LED devices decreases markedly [11], posing great challenges to realize monolithic integration of micro-LED displays as well as visible light communication [12,13]. Besides, the large lattice mismatch between InGaN QWs and GaN quantum barriers (QBs) gives rise to high defect density that degrades the crystalline quality of QWs [14]. The aforementioned factors hinder the development of InGaN-based LEDs simultaneously possessing long wavelength and high output power.

Inspiringly, much effort has been devoted to optimizing the device performance in terms of adopting semi/nonpolar substrate [15], inserting prelayers prior to QWs [16], utilizing bandgap engineering strategy, etc. Benefiting from heteroepitaxial techniques, engineering quantum structures (including QWs [17,18,19], QBs [20,21], and electron-blocking layers [22]) have been reported to effectively promote device performance for the deep ultraviolet and visible emission regions. Engineering QW bandgap, in particular, plays an effective role in confining carriers and enhancing electron-hole wavefunction overlap. By adjusting quantum structural parameters, different QW structures could be realized, such as staggered shape [19,23] and triangular shape [24,25]. Compared to conventional square QWs, LEDs utilizing staggered QWs have demonstrated not only higher light output power (LOP) and alleviated efficiency droop [25], but also better crystalline quality [19]. However, there are relatively few experimental research regarding growth condition modulation for staggered QW structure as well as its application for LEDs, which deserve further exploration to thoroughly understand the related mechanisms behind them.

In this work, we varied TMIn flux to grow a low-In-composition layer (LIL) in staggered InGaN QWs and surprisingly found that, when fixing other growth conditions, decreasing TMIn flux for LIL growth contributes to enhanced LOP as well as longer wavelength emission of LED devices. Based on the experimental data, the strain-induced composition pulling effect is alleviated in staggered QWs because of reduced lattice mismatch between GaN QBs and LIL grown under low TMIn flux, which enables more indium incorporation in the following high-In-composition layer (HIL). Compared to LIL grown at high TMIn flux, surface morphology is improved for LIL grown at low TMIn flux with an atomically flat surface, with root-mean-square roughness of 0.248 nm. Moreover, numerical simulation analysis provides evidence for the unexpected optoelectronic properties of aspects of electron-hole wavefunction overlap and electric field. Our study provides new insights into designing epitaxial structures, which facilitates InGaN-based LEDs towards highly efficient and long-wavelength emission prospects.

## 2. Experiments

**Figure 1:**
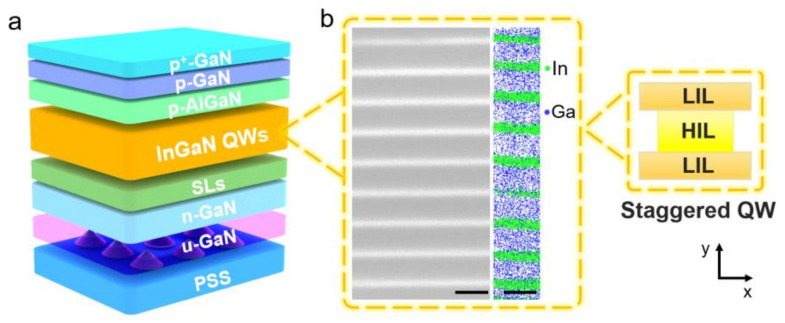
(**a**) Schematic of epitaxial structure of LED based on staggered InGaN QWs. (**b**) Cross-sectional TEM image and APT-reconstructed atom map of QWs in LED epitaxial structure. Scale bar: 20 nm; *x* axis: energy; *y* axis: growth direction.

The LED samples were grown on a *c*-plane patterned sapphire substrate (PSS) using a metal organic chemical vapor deposition (MOCVD) system. As shown in Figure 1a, the LED devices included a 3 µm-thick undoped GaN layer, a 2 µm-thick Si-doped *n*-GaN layer, five pairs of InGaN (1.6 nm)/GaN (8 nm) superlattices (SLs), nine pairs of In_x_Ga_1−x_N (0.6 nm)/In_y_Ga_1−y_N (1.3 nm)/In_x_Ga_1−x_N (0.6 nm)/GaN (14 nm) staggered QWs (x < y), a 25 nm-thick low temperature *p*-GaN layer (not depicted in Figure 1a), a 25 nm-thick AlGaN electron-blocking layer, a 40 nm-thick Mg-doped *p*-GaN layer, and a 2 nm-thick Mg-doped *p*^+^-GaN layer. Notably, TMIn (trimethylindium) flux rate for In_x_Ga_1−x_N LIL growth was set to be 33 sccm, 66 sccm and 100 sccm for LEDs A, B and C, respectively, while other growth conditions were fixed (growth pressure was 200 torr and bubbler temperature of TMIn was 30 °C). Figure 1b displays the cross-sectional bright-field TEM image and the APT-reconstructed atom map of QWs in LED epitaxial structure. QW and QB layers exhibit clear interface, together with the distribution of In and Ga elements.

After epitaxial growth, the standard LED chip fabrication process was carried out. A mesa depth of approximately 1.2 μm was defined to expose the *n*-GaN layer by BCl_3_/Cl_2_ inductively-coupled plasma etching. A 100 nm-thick indium tin oxide (ITO) layer was then deposited on the *p*-GaN layer as a *p*-type Ohmic contact, which was followed by thermal annealing at 350 °C for 30 min in a N_2_ atmosphere. Cr/Pt/Au multilayers were deposited on the ITO and *n*-GaN as *p*- and *n*-type electrodes via e-beam evaporation. Lastly, LED wafers were diced into chips with dimensions of 279 μm × 335 μm.

The cross-sectional transmission electron microscopy (TEM) measurement was performed to characterize the QWs of LED samples. The atom probe tomography (APT) was used by the LEAP 4000X HR system (CAMECA, USA) and the APT data were reconstructed by CAMECA IVAS software (version 3.6.2). The electroluminescence (EL) spectra and peak wavelength versus current and light output power versus current properties were measured using an integrating sphere and semiconductor parameter analyzer. Structural parameters of LED samples were characterized by high-resolution X-ray diffraction (XRD, PANalytical X’Pert Pro MRD diffractometer using Cu Kα1 radiation). The ω—2θ scan based on XRD analysis gave information about the indium composition of InGaN QWs. Scanning electron microscope (SEM) and atomic force microscopy (AFM) were employed to characterize the surface of LED samples.

## 3. Results and Discussion

**Figure 2:**
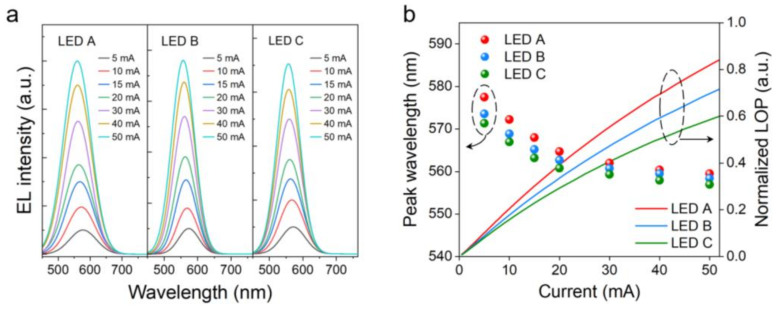
(**a**) Room-temperature EL spectra of LEDs A, B and C as functions of injection currents. (**b**) Normalized LOP and EL peak wavelength as functions of injection currents.

Figure 2a demonstrates the room-temperature EL spectra of LEDs. With the current increasing, peak wavelength for these LED devices shows a blue shift towards the shorter wavelength region, which is attributed to charge screening of the QCSE and band-filling effect [26]. For the hexagonal wurtzite heterostructure of III-nitride materials, QCSE induced by polarization field causes band edge tilting, thus reducing the effective bandgap of QWs and red-shifting the emission wavelength [27]. The injected charges give rise to extra electric field to screen QCSE. Thus, to some extent, blue shift value with increasing current reflects the degree of QCSE caused by intrinsic polarization field in QWs [28]. It is highlighted that LED A exhibits longer-wavelength emission (~580 nm) and larger blue shift of ~18 nm compared to other two LED samples. This indicates that low TMIn flux for LIL is beneficial to indium incorporation for staggered QWs, along with increased polarization field induced-QCSE in QWs. Generally speaking, QCSE has a negative impact on device performance and thus LED A, which suffers from more severe QCSE, should be inferior to LEDs B and C. However, we surprisingly found that LED A shows the highest LOP among these samples, which is approximately 17% and 28% higher than LEDs B and C at 20 mA, respectively, as shown in Figure 2b. It is worth noting that this unexpected observation is based on a large number of experimental devices rather than accidents in the fabrication process shown in Figure S1 (for more experimental details, please refer to Supplementary Materials). When considering the intrinsic difference in staggered QWs from conventional square-shaped QWs, more than one mechanism should account for such unexpected optoelectronic properties, which deserves comprehensive analysis.

**Figure 3:**
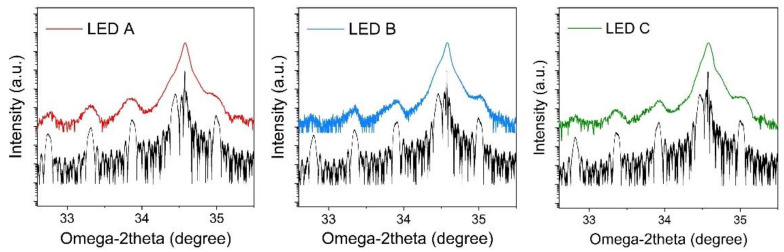
XRD (0002) ω-2θ scan curves for LEDs A, B and C. Black lines represent the fitting curves.

Figure 3 shows the XRD (0002) ω—2θ scans for LED samples with staggered QWs grown under different indium flux. The strongest peak comes from the underlying GaN layers and satellite peaks come from InGaN QWs [29]. In particularly, several satellite peaks can be observed, implying abrupt interfaces between epilayers in QWs achieved. Via fitting the ω—2θ curves with X’pert Epitaxy software, the average indium content in sample was estimated to be ~36%, 34%, and 33% for LED A, B and C, respectively. This implies that LIL grown under low TMIn flux enables indium incorporation in HIL, consistent with the longer wavelength for sample A.

The peak wavelength difference is related to the different indium composition in the HIL of staggered QWs, which essentially derives from the difference in LIL growth condition. Under other fixed growth conditions (growth temperature below 850 °C), increasing TMIn flux has been reported to increase indium composition in QWs grown by MOCVD and metalorganic vapor phase epitaxy techniques [30,31]. In our study, higher indium composition was achieved in LIL with higher indium flux, while accompanied by a relatively larger lattice mismatch between the LIL and GaN layers. This leads to enhanced strain between epilayers, which causes a composition pulling effect [31], further affecting the subsequent HIL growth process. In detail, strain excludes indium atoms to the surface of the InGaN layer to reduce the deformation energy because of the lattice mismatch [32]. A theoretical study revealed that compressive strain suppresses indium incorporation, while tensile strain promotes it [33]; that is to say, HIL grown on LIL with increased indium content suffers from an enhanced composition pulling effect, which makes indium incorporation more difficult. Accordingly, the wavelength difference caused by different TMIn flux is explained.

As for LOP enhancement, there may be two possible factors. On the one hand, improved crystalline quality commonly has a positive influence on the device performance. For the III-nitride material system, different defect types have been previously found to exist in epilayers, i.e., threading dislocation [34], point defect [35], and stacking default [36]. These defects, acting as non-radiative recombination centers, introduce deep levels in the bandgap and prevent carriers from radiative recombination, eventually deteriorating the optoelectronic properties [6,37]. On the other hand, radiative recombination rate is improved by increasing electron-hole wavefunction overlap in QWs, which is an innate advantage of such staggered QWs as introduced above.

**Figure 4:**
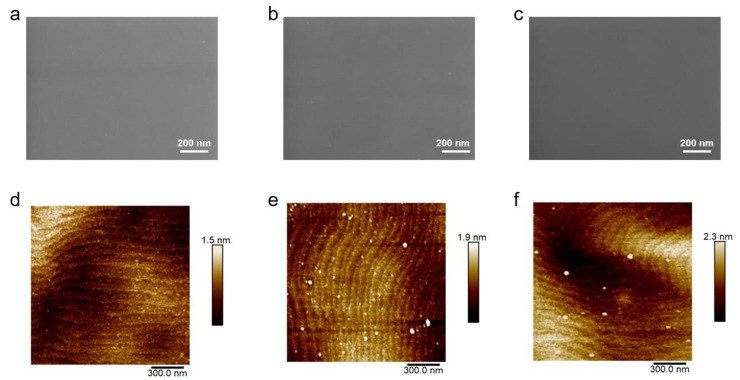
SEM images of LEDs (**a**) A, (**b**) B and (**c**) C. AFM images of LEDs (**d**) A, (**e**) B and (**f**) C.

Figure 4a–c shows the SEM images of the surface morphology of LEDs A–C, respectively. Very flat surface is observed for LED samples, without obvious structure defects that were formed in the underlying layers and extended to the surface. To further examine the morphology and roughness of the sample surface in detail, AFM analysis on an area of 1.5 × 1.5 μm^2^ was performed (Figure 4d–f). All LED samples feature atomically flat surface with parallel steps and terraces microscopic morphology, which is typical for III-nitride epilayers. It was reported that under high TMIn flux rate, adatoms have inadequate time to incorporate into the step edge, thus degrading the surface morphology [38]. In our case, crystalline quality difference in LIL induced by flux rate further affected the following epilayers not limited to HIL. As a result, LED C, whose LIL in staggered QWs was grown under relatively high TMIn flux rate, featured a relatively rough surface with a root-mean-square (RMS) roughness of 0.485 nm, while RMS value for LEDs A and B was 0.248 nm and 0.344 nm, respectively, indicating improvement in crystalline quality with relatively low indium flux for LIL growth.

**Figure 5:**
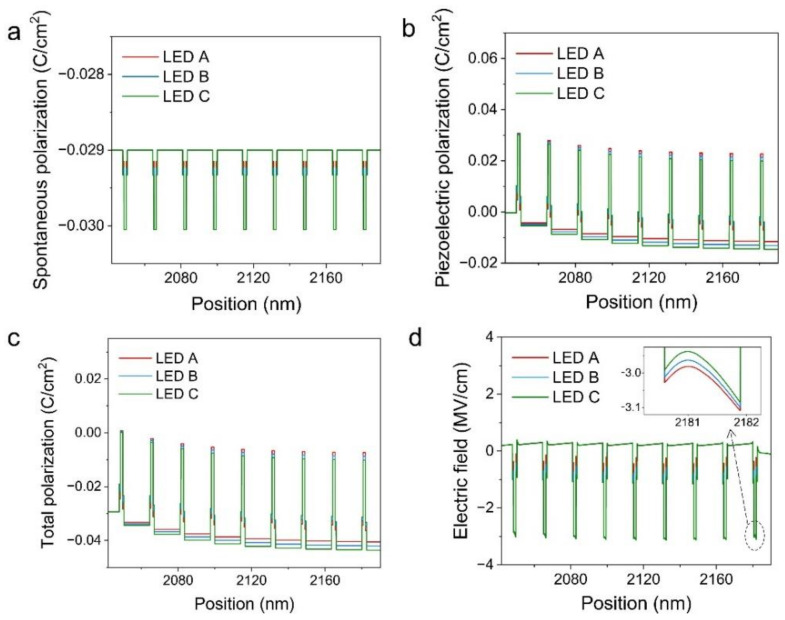
(**a**) Simulated spontaneous polarization fields for LEDs. (**b**) Simulated piezoelectric polarization fields. (**c**) Simulated total polarization fields for LEDs. (**d**) Simulated electric fields for LEDs. The inset in (**d**) shows the enlarged fragment of electric fields in the QW closest to p-side.

SiLENSe simulation software was used to numerically investigate the performance of LEDs based on staggered QWs. The electron and hole mobilities were 100 cm^2^ V^−1^ s^−1^ and 10 cm^2^ V^−1^ s^−1^, respectively. The InGaN/GaN band offset ratio (∆*E*c: ∆*E*v) was set to be 0.7: 0.3. To investigate the effect of LIL on the electric field in HIL, the indium composition in HIL was set to 36%, while that in LIL was set to 5%, 8% and 11% in the simulation for LEDs A, B, and C, respectively. Other parameters of epitaxial structure used in in the simulation were set to be the same as those in fabricated devices. Via numerical simulation, we investigated the influence of indium content in LIL on the polarization electric filed in QWs. Figure 5a–c show the polarization field in QWs of these samples at 20 mA. The electric field profiles of these samples at the injection current of 20 mA are shown in Figure 5d. The intrinsic wurtzite structure of III-nitrides induces spontaneous polarization, while lattice mismatch between epilayers induces piezoelectric polarization [39]. Because of discontinuities in the sum of spontaneous and piezoelectric polarizations between adjacent epilayers, immobile sheet charges of opposite signs are formed at opposite well/barrier and LIL/HIL interfaces [40], which can be expressed by

(1) $${\left(P_{sp}+P_{pz}\right)}_{layer1}-{\left(P_{sp}+P_{pz}\right)}_{layer2}=\sigma_{S}^{Pol}$$

where $P_{sp}$ and $P_{pz}$ are the spontaneous and piezoelectric polarizations, respectively, and $\sigma_{S}^{Pol}$ is the induced sheet charge. Additionally, bulk charge induced by polarization can be expressed by $\rho_{B}^{Pol}\left(z\right)=\nabla \cdot P\left(z\right)$, where ∇ is divergence operator, and *P*(*z*) is the polarization field along the z direction. The polarization-induced electric field (*E_w_*) in QWs and net polarization charge density (ΔP) can be expressed by [41]

(2) $$E_{w}=\frac{l_{b}\Delta P}{l_{b}\cdot \varepsilon_{w}+l_{w}\cdot \varepsilon_{b}}$$

(3) $$\Delta P_{z}={\left.\sigma_{S}^{Pol}\right|}_{z=0}-\rho_{B}^{Pol}\cdot z\;\left(z<l_{w}\right)$$

(4) $$E_{w}\cdot l_{w}+E_{b}\cdot l_{b}=0$$

where *E_b_* is defined as electric field in QBs, and *l_w_* and *l_b_* represent the thickness of the QWs and QBs, respectively. From the simulation results, polarization density varied for staggered QWs with different indium content in LIL, associated with different electric field intensity. According to the aforementioned equations, InGaN LIL with lower indium composition gives rise to a lower polarization difference with GaN QB and thus less $\sigma_{S}^{Pol}$. This eventually leads to lower electric field in LIL. Similarly, for HIL grown on lower indium-composition LIL, there exists a higher polarization-induced sheet charge at LIL/HIL interface, eventually inducing higher electric field in HIL of LED A. As shown in Figure 5d, LED A undergoes enhanced electric field intensity compared to LEDs B and C, which is consistent with the experimental result that LED A exhibits a more obvious blue shift in peak wavelength with injection current. In addition to more indium incorporation, there is stronger electric field in HIL of staggered QWs, therefore causing the emission towards the longer-wavelength region.

**Figure 6:**
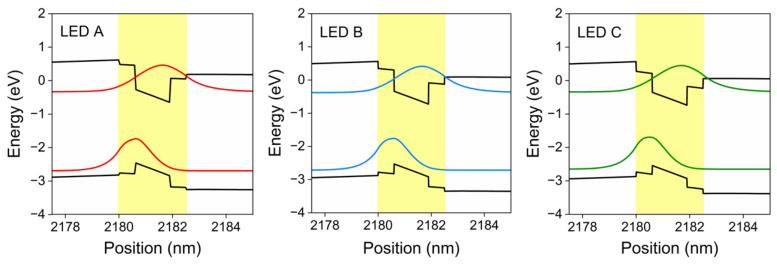
Simulated energy band diagram and carrier wavefunctions of the QW closest to p-side for LEDs A, B and C.

Because holes have larger effective mass and lower mobility compared to electrons, which makes holes mainly distribute in the QWs close to the p-side, we specifically investigated the characteristics of the QW closest to the p-side. Figure 6 shows the simulated energy band profile and carrier wavefunction in QW for LEDs A, B and C, where varying degrees of spatial separation of electron-hole wavefunction can be observed. In general, larger internal electric field makes carrier wavefunction separate towards opposite direction in QWs, degrading quantum efficiency as introduced previously, but LED C shows the lowest wavefunction overlap value of ~30% among these samples. On the contrary, the highest value of ~38% was achieved in LED A. We attribute this to the innate feature of bandgap engineering staggered QWs where LIL structure has a significant influence on regulating carrier confinement, even over the influence caused by electric field. These results also account for the enhanced LOP for LED devices.

## 4. Conclusions

In summary, unexpected simultaneous increases in emission wavelength and LOP was observed in bandgap-engineered staggered InGaN QWs by adjusting TMIn flux rate applied in yellow LED devices. The EL measurement results show that the LIL of staggered QWs grown under low TMIn flux rate contributes to both longer-wavelength emission and higher LOP in fabricated LED devices, as compared to LIL grown under high TMIn flux rate. With the increasing flux rate, more indium composition is obtained in LIL, which aggravates the lattice mismatch between InGaN and GaN layers, leading to increased compressive strain. The composition pulling effect, induced by strain, hinders indium incorporation in HIL and is thus disadvantageous to realize long-wavelength emission. According to AFM analysis, crystalline quality improves with flat surface morphology under growth condition of low flux rate, which partly explains LOP enhancement in LED devices. Moreover, numerical simulation reveals that staggered structure with low flux-rate-grown LIL favors increased carrier wavefunction overlap but underwent enhanced electric field. This finding not only further demonstrates the reason for LOP enhancement, but also accounts for longer-wavelength emission and meanwhile an obvious blue shift by QCSE. Combined with the experimental and numerical results, we conducted systematic and in-depth research on unexpected optoelectronic characteristics in staggered QWs caused by TMIn flux rate, due to both crystalline quality and intrinsic staggered quantum structure. Therefore, our work provides a facile strategy to achieve yellow LEDs with high LOP by bandgap engineering epitaxial growth, eventually to broaden its application in various electronics and optoelectronics.

## Data Availability

The data that support the findings of this study are available from the corresponding author upon reasonable request.

## Supplementary Materials

The following supporting information can be downloaded at: https://www.mdpi.com/article/10.3390/nano12193378/s1, Figure S1: Normalized LOP and EL peak wavelength at 20 mA for samples grown under different TMIn flux. Reference [42] was cited in the Supplementary Materials.
