# Supplementary material for: Unexpectedly Simultaneous Increase in Wavelength and Output Power of Yellow LEDs Based on Staggered Quantum Wells by TMIn Flux Modulation
Source: Nanomaterials (Basel). 2022 Sep 27;12(19):3378. doi: 10.3390/nano12193378 (PMC9565747; doi:10.3390/nano12193378)
Supplement: Supplementary file 1 [file nanomaterials-12-03378-s001.zip › nanomaterials-1861669-SM.pdf]

nanomaterials

Supplementary Materials

Unexpectedly Simultaneous Increase in Wavelength and Output Power of Yellow LEDs Based on Staggered Quantum Wells by TMIn Flux Modulation

Zhenxing Lv [1,†], Xiaoyu Zhao [1,†], Yuechang Sun [1], Guoyi Tao [2], Peng Du [1] and Shengjun Zhou [1,*]

1 Center for Photonics and Semiconductors, School of Power and Mechanical Engineering, Wuhan University, Wuhan 430072, China
2 The Institute of Technological Sciences, Wuhan University, Wuhan 430072, China
* Correspondence: zhousj@whu.edu.cn
† These authors contributed equally to this work.

S1. Results and Discussion

Figure S1 shows the measured LOP-peak wavelength data of LED devices whose quantum wells were grown by different TMIn flux. Specifically, samples grown under 33, 66 and 100 sccm are named as samples A, B and C, respectively. For each sample, more than 30,000 LED devices were fabricated based on the corresponding samples. Moreover, LEDs A, B and C were fabricated based on samples A, B and C, respectively. Although LOP-wavelength results vary for samples grown under specific TMIn flux, resulting from the composition fluctuation [1], it can be seen that samples grown under relatively lower TMIn flux show simultaneously increased wavelength and LOP. This observation is different from the previous experience that LED performance deteriorates markedly with the increasing emission wavelength, confirming that the observed "unexpected" behavior does exist, not by accident.

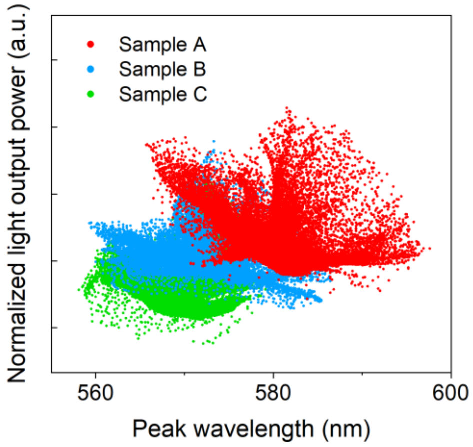

Figure S1. Normalized LOP and EL peak wavelength at 20 mA for samples grown under different TMIn flux.

Reference

1. Okamoto, K.; Kaneta, A.; Kawakami, Y.; Fujita, S.; Choi, J.; Terazima, M.; Mukai, T. Confocal microphotoluminescence of InGaN-based light-emitting diodes. *J. Appl. Phys.* **2005**, *98*, 064503. https://doi.org/10.1063/1.2037869.
